# Sustainable Development of Sawdust Biochar as a Green and Promising Material for CO_2_ Capture Technologies

**DOI:** 10.3390/ma18143243

**Published:** 2025-07-09

**Authors:** Ki-Seok Kwon, Han-Seung Lee

**Affiliations:** 1 Department of Smart City Engineering, Hanyang University, 1271 Sa 3-dong, Sangnok-gu, Ansan-si 15588, Republic of Korea; kskwonn@naver.com; 2 Department of Architectural Engineering, Hanyang University, 1271 Sa 3-dong, Sangnok-gu, Ansan-si 15588, Republic of Korea

**Keywords:** biochar, CO_2_ storage, porosity, BET surface area, carbon capture and storage (CCS), chemical activation, microporosity

## Abstract

This study investigates the synthesis of highly porous ZnCl_2_-activated biochars derived from sawdust through controlled pyrolysis at 300 °C and 500 °C, aiming to enhance CO_2_ adsorption performance. The effects of pyrolysis temperature and chemical activation on particle size distribution, surface area, and pore structure are systematically analyzed. Particle size analysis reveals that higher pyrolysis temperature and ZnCl_2_ activation significantly reduce both median and mean particle sizes, resulting in finer and more uniform biochar morphology. BET analysis demonstrates a substantial increase in specific surface area and micropore volume upon ZnCl_2_ activation, particularly at 500 °C, where the activated biochar (S500ZC) exhibits a high surface area of 717.60 m^2^/g and a micropore area of 616.60 m^2^/g. CO_2_ adsorption isotherms recorded at 25 °C confirm that both thermal treatment and activation markedly enhance adsorption capacity, with the highest uptake of 35.34 cm^3^/g achieved by S500ZC. The adsorption performance follows the order: S300NZC < S300ZC < S500NZC < S500ZC, closely correlating with microporosity and surface textural development. The findings highlight the potential of ZnCl_2_-activated biochars as cost-effective, environmentally friendly, and efficient sorbents for scalable CO_2_ mitigation technologies.

## 1. Introduction

Emissions of carbon dioxide (CO_2_) from human activities, especially the burning of fossil fuels and diverse industrial processes, are largely acknowledged as a significant factor in climate change [1,2]. Several studies have indicated that atmospheric CO_2_ levels have been consistently rising since the industrial revolution, leading to global temperature increases, ocean acidification, and ecological disturbances [3,4]. The ongoing dependence on coal, oil, and natural gas, particularly in electricity generation, transportation, and heavy industries like cement and steel production, has resulted in a significant rise in greenhouse gas emissions [5]. Global climate organizations and scientific entities have consistently highlighted that, without urgent and ongoing mitigation actions, the effects of increasing CO_2_ levels will become progressively more severe [6,7]. This critical scenario highlights the necessity for efficient approaches to control and lower CO_2_ emissions in different sectors.

Technologies for carbon capture and storage (CCS) have become an essential method to tackle these issues [7]. CCS consists of capturing CO_2_ at its origin, compressing and transporting it, and then either storing it in geological formations or using it in industrial applications [8]. It is commonly seen as a vital instrument in the collection of strategies designed to reach net-zero emissions. Among the different CO_2_ capture techniques, post-combustion capture is the most commonly applicable, especially for modifying existing fossil fuel-powered plants [9,10]. Nevertheless, traditional technologies like amine-based solvents, while efficient, face various drawbacks such as significant energy use for regeneration, corrosion problems, deterioration after multiple cycles, and environmental issues related to solvent disposal [11,12]. These limitations have prompted investigations into other solid sorbents capable of providing significant CO_2_ absorption, selectivity, and thermal and chemical resilience, along with minimal regeneration expenses.

Biochar, a carbon-rich porous substance produced from the thermal transformation of biomass in low-oxygen environments, has gained growing interest as an eco-friendly material for CO_2_ adsorption [13,14,15]. Generated from diverse organic materials like agricultural byproducts, forestry waste, livestock manure, and sewage sludge, biochar provides a sustainable, affordable, and eco-friendly choice for carbon sequestration [1,16]. The pyrolysis method not only prevents waste from entering landfills but also produces a stable carbon-rich material that can remain in the environment for hundreds to thousands of years. The inherent stability of carbon in biochar makes it a vital resource for sequestering carbon [17,18]. Moreover, the physicochemical characteristics of biochar, like elevated surface area, adjustable porosity, and surface functional groups, can be modified to improve its engagement with CO_2_ molecules [19,20,21].

In contrast to traditional sorbents like zeolites and metal–organic frameworks (MOFs), which often involve intricate synthesis and high costs, biochars can be created through comparatively straightforward and scalable methods. Biochar characteristics can be modified by choosing appropriate feedstocks and altering pyrolysis conditions like temperature, heating rate, and duration [22]. Moreover, chemical activation (e.g., using KOH, ZnCl_2_, H_3_PO_4_) and surface functionalization (e.g., nitrogen doping, amine grafting) are commonly utilized to further improve the adsorption capacity and selectivity of biochars for CO_2_ [23]. Such changes usually lead to a rise in microporosity and the addition of basic functional groups that positively interact with acidic CO_2_ molecules [24]. Certain engineered biochars have shown CO_2_ adsorption capabilities surpassing 2 mmol/g under ambient conditions, positioning them as rivals to commercial activated carbons and various solid adsorbents [11].

An additional important benefit of biochar is its sustainability and circularity. Using waste biomass as a raw material, biochar production promotes the concepts of waste valorization and circular economy [25]. Additionally, combining biochar-based CO_2_ capture with renewable or low-emission energy systems can yield synergistic environmental advantages, especially when the pyrolysis process is fine-tuned for energy recovery and co-product creation. In some instances, the production of biochar may actually be deemed carbon-negative, based on the lifecycle emissions and the applications at the end of its use [18]. Nonetheless, challenges persist in comprehending the relationships among structure, properties, and performance that dictate CO_2_ adsorption, along with the need for standardized methods to assess and compare biochars created under varying conditions. Additional studies are required to enhance activation techniques, boost adsorption kinetics and selectivity, and guarantee the durability of biochars in real-world operating circumstances.

This study explores the development of sustainable and efficient CO_2_ adsorbents by valorizing sawdust, a widely available lignocellulosic biomass waste, into activated biochar. The primary aim is to investigate how thermal treatment and chemical activation influence the structural properties and CO_2_ capture performance of the resulting materials. Sawdust-derived biochars are synthesized via pyrolysis at two temperatures, 300 °C and 500 °C, to examine how varying degrees of carbonization affect porosity, surface functionality, and adsorption behavior. The lower pyrolysis temperature (300 °C) typically produces biochars with a higher yield and more abundant oxygen-containing functional groups, which can contribute to CO_2_ adsorption through physisorption and weak surface interactions. In contrast, pyrolysis at 500 °C promotes extensive devolatilization and structural aromatization, leading to enhanced microporosity, increased surface area, and higher carbon content, all favorable characteristics for improved CO_2_ uptake. By comparing these two temperature regimes, this work aims to elucidate the role of thermal processing in tailoring the physicochemical properties of biochar for gas adsorption applications.

To further enhance the adsorption capacity, ZnCl_2_ has been introduced as a chemical activating agent prior to pyrolysis. ZnCl_2_ is a well-known Lewis acid with strong dehydrating properties, facilitating the breakdown of hemicellulose and cellulose during pyrolysis while simultaneously suppressing tar formation. This process fosters the development of well-defined micropores and significantly increases surface area—two critical factors governing gas adsorption efficiency. Moreover, ZnCl_2_ activation may induce the formation of oxygenated and heteroatom-containing surface functional groups, which are beneficial for acid-base interactions with CO_2_ molecules. The selection of ZnCl_2_ is motivated not only by its effectiveness in pore development but also by its relative sustainability and ease of recovery compared to other chemical activators like KOH. The combination of controlled thermal treatment and ZnCl_2_ activation may offer a practical pathway to engineer biochar structures optimized for CO_2_ capture. Ultimately, this research aims to contribute a scalable and environmentally benign strategy for carbon mitigation, supporting the global transition toward sustainable materials and circular waste management in climate change mitigation technologies.

## 2. Materials and Methods

### 2.1. Production of Biochar

In this study, sawdust collected from a woodworking shop in Ansan, Republic of Korea, is used as the raw biomass for biochar production. The biomass is oven-dried at 105 °C for 48 h to remove inherent moisture and is then ground using an electric grinder (Cuckoo Electronics, Seoul, Republic of Korea) to obtain a uniform particle size. The ground biomass is divided into two portions: one is subjected to chemical activation using a 1 M aqueous ZnCl_2_ (DUKSAN Reagents, Ansan, Republic of Korea) solution, while the other is kept unmodified to serve as a control. For the activation, 1 kg of biomass is mixed with 1 L of ZnCl_2_ solution and kept for 12 h at room temperature to ensure proper impregnation. A 1 M ZnCl_2_ concentration with 12 h impregnation ensures effective activation by promoting sufficient diffusion into the biomass without structural collapse, enabling uniform pore development during pyrolysis.

Both the activated and non-activated biomass samples are pyrolyzed at two different temperatures, 300 °C and 500 °C, in an electric muffle furnace (Model MSF-22, Lab House, Ansan, Gyeonggi-do, Republic of Korea). The temperature is increased at a constant heating rate of 10 °C/min and then held isothermally at the target temperature for 1 h to complete carbonization. After pyrolysis, the ZnCl_2_-activated biochars are thoroughly washed with deionized water to remove residual ZnCl_2_ and subsequently dried. The resulting biocharsare labeled based on their pyrolysis temperature and activation status: S300NZC (300 °C, non-activated), S300ZC (300 °C, ZnCl_2_-activated), S500NZC (500 °C, non-activated), and S500ZC (500 °C, ZnCl_2_-activated).

### 2.2. Characterizations

The particle size distribution of the biochars is analyzed using a laser scattering particle size distribution analyzer (LA-960, HORIBA, Kyoto, Japan). The surface morphology and elemental composition are examined using a field emission scanning electron microscope (FE-SEM, SU5000, Hitachi, Tokyo, Japan) equipped with an energy-dispersive X-ray spectroscopy (EDS) system. To identify surface functional groups, Fourier-transform infrared spectroscopy (FT-IR; UATR Two, PerkinElmer, Shelton, CT, USA) is employed.

The pore structure and specific surface area are determined by nitrogen adsorption–desorption analysis using the Brunauer–Emmett–Teller (BET) method on an accelerated surface area and porosimetry system (ASAP 2460, Version 3.01, Micromeritics, Norcross, GA, USA). Prior to analysis, the biochar samples are degassed at 150 °C for 6 h. Measurements are conducted at −195.850 °C across a relative pressure (P/P_0_) range of 0.0 to 1.0.

To assess the CO_2_ capture capacity, CO_2_ adsorption–desorption measurements are carried out using a CO_2_ adsorption isotherm analyzer (TriStar II Plus, Version 3.03, Micromeritics, Norcross, GA, USA), also based on the BET method. Before testing, the samples are degassed under vacuum at 110 °C for 10 h. The analysis is performed at a constant bath temperature of 25 °C over a relative pressure (P/P_0_) range from 0.0 to 0.016.

Statistical Analysis: All measurements are conducted in triplicate, and the results are presented as mean ± standard deviation to verify the reproducibility of the experimental procedures, including BET surface area, micropore area, and particle size parameters. Statistical analysis is performed using OriginPro 2023 software to ensure data reliability.

## 3. Results

### 3.1. Particle Size Distribution of Biochar

The particle size distribution of the synthesized biochar samples, as summarized in Table 1, reveals significant changes influenced by both pyrolysis temperature and ZnCl_2_ activation. These factors play a critical role in modifying the physical morphology of biochar particles, thereby impacting their surface area, reactivity, and performance in various applications. An increase in pyrolysis temperature from 300 °C to 500 °C has resulted in a marked reduction in both median and mean particle sizes for the non-activated and ZnCl_2_-activated samples. For non-activated biochar, the median particle size is decreased from 11.489 μm (S300NZC) to 5.704 μm (S500NZC), and the mean particle size is reduced from 17.334 μm to 11.494 μm. A similar trend is observed for ZnCl_2_-activated biochars, where the median size has been decreased from 8.444 μm (S300ZC) to 5.749 μm (S500ZC), and the mean size from 9.461 μm to 8.052 μm. These reductions in particle dimensions are primarily attributed to enhanced thermal degradation and devolatilization processes at higher temperatures. Elevated pyrolysis temperatures accelerate the breakdown of biomass polymers such as cellulose, hemicellulose, and lignin, resulting in fragmentation and pore collapse. This leads to the formation of smaller, denser, and more homogeneous carbon particles.

The incorporation of ZnCl_2_ as an activating agent has further intensified the size reduction at both temperature levels. At 300 °C, ZnCl_2_ activation has caused the mean particle size to decrease significantly from 17.334 μm (S300NZC) to 9.461 μm (S300ZC). At 500 °C, a similar reduction is observed from 11.494 μm (S500NZC) to 8.052 μm (S500ZC). The chemical activation process promotes depolymerization and enhances porosity by facilitating the removal of volatile matter and inhibiting tar formation. ZnCl_2_, a Lewis acid, exhibits a strong dehydrating effect that disrupts hydrogen bonding and weakens the cellulose–lignin matrix during pyrolysis, thereby promoting finer particle formation through structural disintegration.

Moreover, the cumulative size distribution (D_10_, D_50_, and D_90_ values) confirms a general narrowing of particle size distribution with increasing temperature and activation. Notably, the D_90_ value, a measure of the diameter below which 90% of the particles fall, decreased from 33.330 μm (S300NZC) to 25.913 μm (S500NZC) in non-activated samples, and from 15.299 μm (S300ZC) to 14.589 μm (S500ZC) in activated samples. This reduction in D_90_ indicates a substantial decline in the fraction of large particles and reflects an improvement in particle size uniformity. The D_10_ values also reduced (e.g., from 4.387 μm in S300NZC to 1.616 μm in S500NZC), suggesting that a larger proportion of ultra-fine particles formed under higher severity conditions. In addition to particle size, optical transmittance data also suggest morphological evolution. The red and blue channel transmittance values progressively declined with increasing temperature and activation, suggesting denser and optically darker biochar particles, likely due to increased graphitization and aromatic condensation.

### 3.2. Scanning Electron Microscopy and Energy-Dispersive X-Ray Spectroscopy

**Figure 1:**
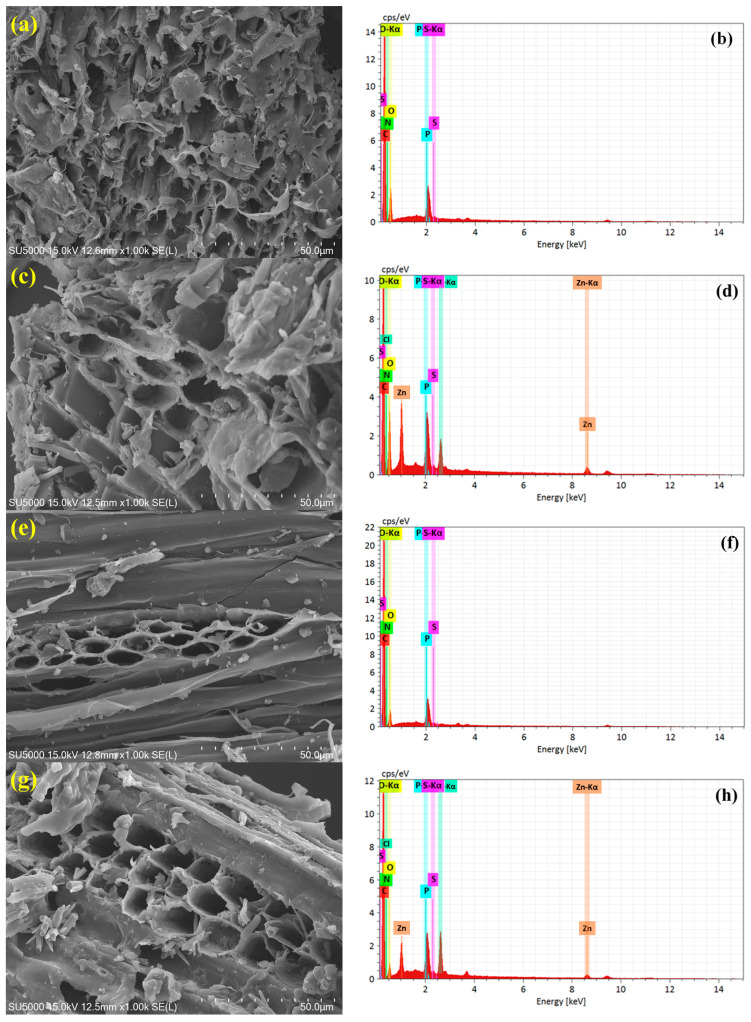
Surface morphology by FE-SEM and respective EDS spectra of (**a**,**b**) S300NZC, (**c**,**d**) S300ZC, (**e**,**f**) S500NZC, and (**g**,**h**) S500ZC.

The surface morphology and elemental composition of the synthesized biochars are systematically analyzed using SEM-EDS, as illustrated in Figure 1a–h. The SEM micrographs clearly show that both the pyrolysis temperature and ZnCl_2_ activation play pivotal roles in tailoring the surface architecture of the biochars. At 300 °C, the non-activated biochar (S300NZC) exhibits a relatively dense, compact structure with minimal porosity, indicating partial thermal degradation of the biomass. In contrast, the ZnCl_2_-activated S300ZC shows a moderately developed porous texture, with evident surface etching and early-stage pore formation due to the dehydrating and foaming effect of ZnCl_2_ during pyrolysis. However, these morphological changes become significantly more pronounced at 500 °C. The non-activated S500NZC displays an aligned, tubular structure, likely retaining partially intact vascular bundles of the precursor biomass. Remarkably, the activated S500ZC exhibits a well-developed, interconnected, and sponge-like microporous framework with thin walls and abundant voids, indicative of enhanced carbon skeleton reorganization and volatile matter removal facilitated by ZnCl_2_ activation at higher temperature [26].

To complement the surface morphology, EDS spectra and elemental analysis (Table 2) provide vital information on the compositional evolution of the biochars under varying pyrolysis and activation conditions. Carbon (C), nitrogen (N), and oxygen (O) dominate the elemental composition, with phosphorus (P) and sulfur (S) present as minor yet functional heteroatoms. Compared to the non-activated samples, the ZnCl_2_-treated biochars (S300ZC and S500ZC) exhibit notable levels of residual Zn (3.23% and 1.75%) and Cl (1.67% and 1.53%), respectively. These values indicate that a small fraction of Zn and Cl remains post-washing, though their presence does not significantly influence the CO_2_ adsorption mechanism under investigation.

If the weight percentages of key elements are considered without Zn and Cl, the C, N, and O contents in S300ZC are 51.2%, 10.2%, and 29.1%, respectively, while in S500ZC, they are 64.5%, 15.3%, and 12.4%, respectively. These values highlight that ZnCl*_2_* activation leads to a modest reduction in oxygen content and a relative increase in nitrogen and carbon. Interestingly, the nitrogen content remains notably high in the S500 samples (15.3–15.8 wt.%), reflecting the thermal stability of nitrogen-containing functional groups such as pyridinic-N and graphitic-N. These species are known to enhance CO*_2_* adsorption through increased active sites and improved electronic conductivity. Similarly, ZnCl_2_ activation promotes phosphorus incorporation, as evident from the elevated P content in S300ZC (4.2%) and S500ZC (4.1%) compared to their non-activated counterparts. The presence of phosphorus may be attributed to partial retention or surface deposition during pyrolysis. Sulfur, although present in small amounts (0.1–0.4 wt.%), is retained more effectively in activated samples and may contribute to surface redox activity and CO*_2_* interactions [27].

Elemental mapping from EDS confirms the uniform distribution of C, N, O, P, and S across the carbon matrix, suggesting homogenous presence rather than localized clustering. Such uniform heteroatom existence is essential for tailoring surface polarity, introducing defect sites, and enhancing adsorption, catalytic, or electrochemical behaviors. Collectively, these results emphasize that the synergistic effects of optimized pyrolysis temperature and ZnCl_2_ activation result biochars with highly porous morphologies, high carbon content, and effective heteroatom doping, rendering them excellent candidates for advanced applications such as gas adsorption, energy storage, and catalysis [28].

### 3.3. FTIR Spectroscopy

**Figure 2:**
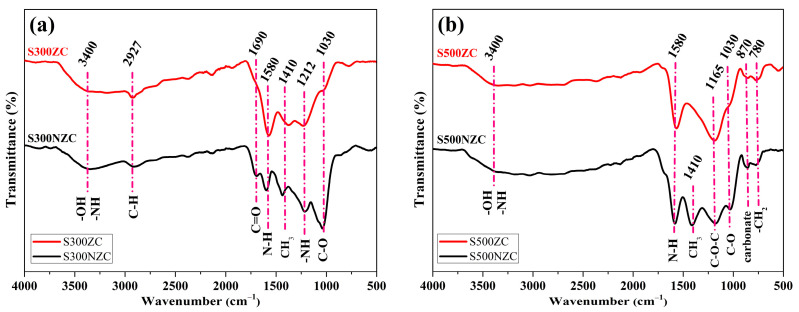
FTIR spectra of (**a**) S300NZC and S300ZC, and (**b**) S500NZC and S500ZC.

The FTIR spectra of the biochar samples presented in Figure 2a,b provide insightful information regarding the evolution of surface functional groups as influenced by pyrolysis temperature and ZnCl_2_ activation. Across all samples, a broad absorption band centered around 3400 cm^−1^ is observed, corresponding to the stretching vibrations of hydroxyl (–OH) and amine (–NH) groups [29,30], which are commonly derived from lignocellulosic biomass. This feature confirms the retention of polar functionalities that can influence hydrophilicity and surface reactivity. A weak but distinguishable peak at 2927 cm^−1^, attributed to C–H stretching vibrations in aliphatic hydrocarbons, appears in both S300NZC and S300ZC [31,32]. However, this peak disappears in the 500 °C samples Figure 2b, indicating the effective decomposition of thermally unstable aliphatic chains and volatile organic moieties under higher pyrolysis temperatures.

The absorption band at 1690 cm^−1^, associated with the stretching vibrations of carbonyl (C=O) groups in carboxylic acids or ketones [33,34], is clearly evident in S300NZC but diminishes in the ZnCl_2_-activated S300ZC and completely vanishes in both 500 °C samples. This observation highlights the progressive loss of carboxyl functionalities with increasing temperature, consistent with enhanced decarboxylation and aromatization processes. A prominent and persistent peak around 1580 cm^−1^, assigned to N–H bending and/or skeletal vibrations of aromatic rings [35,36], is present in all samples irrespective of treatment. This suggests the thermal stability of certain nitrogenous structures or aromatic domains, which are more resilient during pyrolysis and may play a crucial role in tuning adsorption and electrochemical properties.

Another noticeable feature is the band at 1410 cm^−1^, attributed to –CH_3_ bending vibrations [37]. This peak remains visible across all samples, although its relative intensity decreases at higher temperatures, indicating partial cleavage of methyl-substituted structures. In the 300 °C samples, particularly S300ZC, a shoulder at 1212 cm^−1^ is observed, corresponding to N–H wagging modes typically found in primary or secondary amines [37]. This peak disappears entirely in both 500 °C samples, pointing to the thermal degradation of labile nitrogen functionalities during high-temperature treatment.

The band at 1030 cm^−1^, representative of C–O stretching vibrations from alcohols, ethers, or ester groups [38], appears across all samples but is significantly reduced in intensity for ZnCl_2_-activated specimens. This suppression of oxygenated functionalities suggests partial chemical etching and dehydration induced by the Lewis acidic nature of ZnCl_2_, which facilitates oxygen removal and promotes structural rearrangement. Importantly, at 500 °C, especially in the S500ZC sample, several new features emerge: a distinct peak at 1165 cm^−1^ corresponding to asymmetric stretching of C–O–C linkages [39], a sharp peak at 870 cm^−1^ attributed to carbonate groups [35,40], and a smaller band at 780 cm^−1^ arising from CH_2_ bending vibrations [41,42]. The presence of these bands indicates increased mineralization and formation of stable structural motifs during high-temperature pyrolysis, further promoted by the catalytic activation effects of ZnCl_2_.

The FTIR analysis reveals that elevated pyrolysis temperature facilitates the removal of labile organic and nitrogen-containing groups, while ZnCl_2_ activation significantly alters the oxygenated functionalities, leading to structural reorganization and enhanced aromaticity. The persistence of nitrogen-associated peaks, especially the consistent 1580 cm^−1^ band in S500ZC, suggests that thermally stable nitrogen species remain embedded within the carbon framework. These moieties may contribute to improved CO_2_ adsorption performance due to favorable acid-base interactions with the quadrupolar CO_2_ molecules. The synergistic effect of temperature and chemical activation thus optimizes the surface chemistry of the biochar, enhancing its potential for environmental and energy applications.

The activation mechanism of ZnCl_2_ in the formation of porous biochar structures involves multiple synergistic chemical processes that operate at the molecular level. ZnCl_2_ is a strong Lewis acid that facilitates the catalytic dehydration of lignocellulosic components (cellulose, hemicellulose, and lignin) by coordinating with the oxygen atoms in hydroxyl (–OH), carboxyl (–COOH), and ether linkages [43,44]. This coordination weakens the bonds and promotes the elimination of water molecules at lower thermal thresholds. During pyrolysis, ZnCl_2_ penetrates the biomass matrix and promotes intramolecular cross-linking and aromatization through a charring mechanism, stabilizing the carbon framework and enhancing the structural rigidity. Moreover, Zn^2*+*^ ions interact electrostatically with the electron-rich functional groups, such as phenolics and carboxylates, contributing to the breakdown of large macromolecular assemblies into smaller volatile fragments, which are expelled, leaving behind a microporous carbon skeleton.

### 3.4. Surface Area and Pore Structure

**Figure 3:**
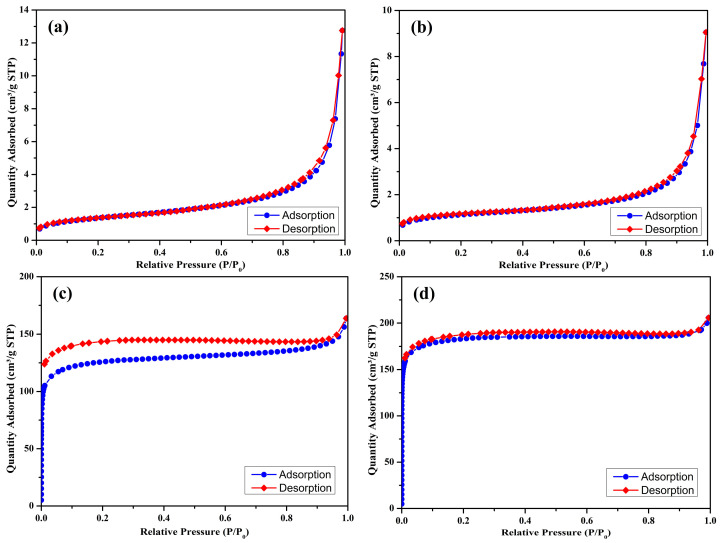
N_2_ adsorption–desorption isotherms (at 77 K) of S300NZC (**a**), S300ZC (**b**), S500NZC (**c**), S500ZC (**d**).

**Figure 4:**
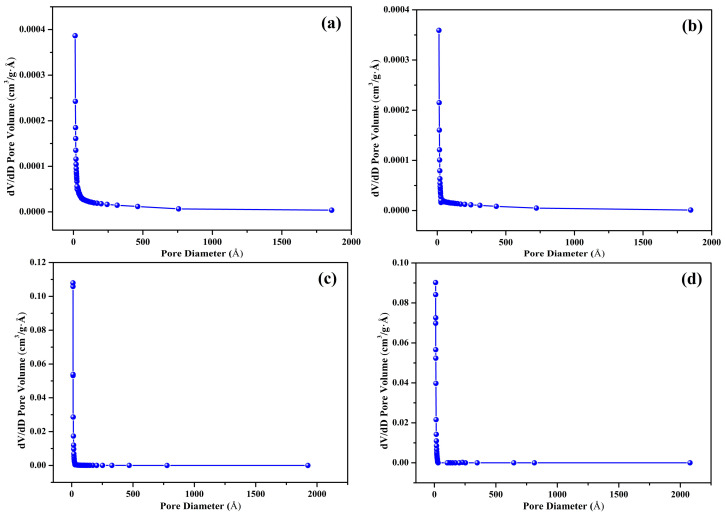
Pore size distribution by BJH method: (**a**) S300NZC, (**b**) S300ZC, (**c**) S500NZC, and (**d**) S500ZC.

The nitrogen adsorption–desorption isotherms of the prepared biochar samples (S300NZC, S300ZC, S500NZC, and S500ZC) are presented in Figure 3a–d, while the corresponding BJH pore size distribution plots are shown in Figure 4a–d. These characterizations provide comprehensive insights into the surface area, porosity, and adsorption behavior of the biochars as influenced by pyrolysis temperature and ZnCl_2_ activation. The quantitative BET surface area, micropore area, and BJH pore diameter values are summarized in Table 3.

S300NZC and S300ZC (Figure 3a,b), pyrolyzed at 300 °C, exhibit Type III isotherms based on the IUPAC classification. These curves, characterized by gradual increases in nitrogen uptake with increasing relative pressure, suggest weak interactions between the adsorbate and adsorbent, typical of nonporous or macroporous materials. The isotherms lack sharp inflection points and display limited hysteresis, further confirming minimal micropore development. Correspondingly, the BET surface areas are very low, 4.96 m^2^/g for S300NZC and 4.12 m^2^/g for S300ZC. However, ZnCl_2_ activation modestly improves the micropore area (from 0.21 m^2^/g to 1.33 m^2^/g), indicating some enhancement in pore formation due to partial chemical etching of the biomass matrix. The BJH pore diameters for both samples remain high, 90.17 Å for S300NZC and 86.06 Å for S300ZC, confirming the predominance of meso to macropores. These characteristics are further validated by the BJH plots in Figure 4a,b, which show broad and poorly defined pore size distributions.

In contrast, the isotherms of S500NZC and S500ZC (Figure 3c,d), obtained at 500 °C, exhibit clear Type I(b) characteristics with a sharp increase in nitrogen uptake at low relative pressures (P/P_0_ < 0.1), indicating the dominance of microporous structures. A pronounced plateau at higher pressures reflects the saturation of micropores, and the significant hysteresis suggests the presence of some mesopores. The BET surface areas increase drastically, reaching 458.14 m^2^/g for S500NZC and 717.60 m^2^/g for S500ZC. These increases are largely attributable to the elevated micropore areas, 365.09 m^2^/g and 616.60 m^2^/g, respectively, resulting from the combined effects of higher pyrolysis temperature and ZnCl_2_ activation. The BJH pore diameters decrease substantially to 15.58 Å (S500NZC) and 14.13 Å (S500ZC), indicating enhanced pore confinement. The BJH plots in Figure 4c,d display narrower and more well-defined peaks, further affirming the development of a uniform microporous structure.

ZnCl_2_ activation plays a minimal role at 300 °C, where limited devolatilization and structural rearrangement occur. However, at elevated pyrolysis temperatures (e.g., 500 °C), ZnCl_2_ can also intercalate between carbon layers and induce swelling, expanding the interlayer spacing and promoting graphitic sheet delamination. Upon post-pyrolysis washing, Zn-containing residues are leached out, resulting in the formation of well-developed micropores and enhanced surface roughness. These structural changes are evident from the significant increases in specific surface area (up to 717.60 m^2^/g) and micropore area (616.60 m^2^/g) observed in S500ZC. Therefore, ZnCl_2_ acts not only as a chemical activator but also as a pore-directing agent that engineers the biochar’s internal architecture for optimal adsorption performance.

The data clearly show that temperature is a critical factor. Without sufficient thermal energy, the structural transformation necessary for micropore development remains incomplete, as seen in the low surface area of the 300 °C samples. Only when pyrolysis is performed at 500 °C does ZnCl_2_ activation reach its full potential in producing biochars with ideal characteristics for gas-phase adsorption. The remarkable increase in surface area and microporosity, particularly in S500ZC, demonstrates the importance of synergistic thermal and chemical activation for developing efficient adsorbents. The high BET surface area (717.60 m^2^/g) and micropore dominance in S500ZC make it especially suitable for CO_2_ adsorption, where the size and accessibility of adsorption sites are critical. The well-developed micro- and narrow mesopores shown in the BJH plots (Figure 4) further support this, as these pore structures are optimal for trapping CO_2_ molecules via physisorption.

### 3.5. CO_2_ Adsorption Characteristics

**Figure 5:**
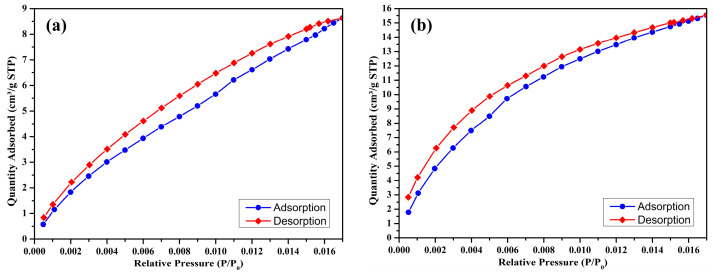

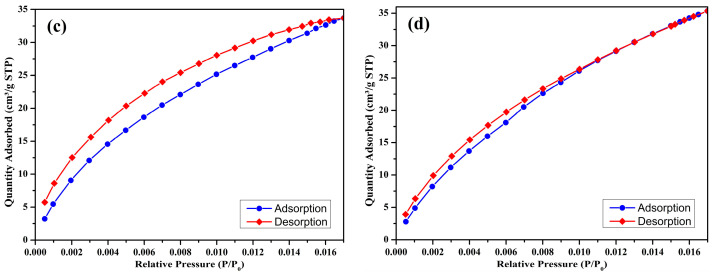
CO_2_ adsorption isotherm (at 298 K) of S300NZC (**a**), S300ZC (**b**), S500NZC (**c**), S500ZC (**d**).

The CO_2_ adsorption–desorption isotherms of the prepared biochar samples are illustrated in Figure 5a–d. These isotherms, recorded at 25 °C, over a low relative pressure range (P/P_0_: 0.000–0.016), demonstrate the physisorption characteristics of CO_2_ on the porous surfaces of the biochars. The CO_2_ adsorption capacity data for each sample is summarized in Table 4.

At a pyrolysis temperature of 300 °C, the non-activated biochar (S300NZC) exhibits a CO_2_ adsorption capacity of 8.691 cm^3^/g STP. When the same biochar is activated with ZnCl_2_ (S300ZC), the CO_2_ uptake increases substantially to 15.5306 cm^3^/g STP. This enhancement reflects the improved microporosity resulting from chemical activation, consistent with the corresponding increase in micropore area shown in the BET analysis. The isotherms for these two samples (Figure 5a,b) display a gradual increase in adsorption with rising pressure, indicative of a less developed microporous structure with limited high-energy adsorption sites.

In contrast, the biochars prepared at 500 °C show significantly higher CO_2_ adsorption capacities. S500NZC adsorbs 33.696 cm^3^/g STP, while S500ZC achieves the highest capacity at 35.3396 cm^3^/g STP. The sharp rise in adsorption performance at this temperature can be directly attributed to the development of extensive microporosity, as confirmed by nitrogen adsorption data and the BET surface area analysis. Notably, S500ZC, which has the highest surface area (717.60 m^2^/g) and micropore area (616.60 m^2^/g), also possesses the smallest average pore diameter (14.13 Å), making it particularly suitable for CO_2_ adsorption due to its proximity to the kinetic diameter of CO_2_ molecules (~3.3 Å).

These results reveal the synergistic effects of ZnCl_2_ activation and elevated pyrolysis temperature on enhancing CO_2_ capture performance. At 300 °C, activation alone improves adsorption by creating additional micropores. However, the most significant impact is observed at 500 °C, where both thermal decomposition and chemical activation work together to generate a highly porous and adsorptive structure. The isotherms of the 500 °C samples (Figure 5c,d) show a steeper rise at low relative pressures, highlighting a strong interaction between CO_2_ molecules and the narrow micropores, an essential characteristic for efficient adsorption at ambient conditions.

The CO_2_ adsorption behavior observed follows the trend S300NZC < S300ZC < S500NZC < S500ZC, which aligns well with the progression in pore structure and surface area. The dominance of microporosity in CO_2_ uptake is evident, as the increase in total surface area and micropore contribution leads to enhanced physisorption. While mesopores facilitate gas transport within the structure, micropores serve as the primary adsorption sites due to their favorable dimensions for gas molecule entrapment and interaction. This observation is further supported by the BET and BJH data, as well as the shape of the isotherms.

The CO_2_ adsorption capacities of the biochars synthesized in this study are compared with other reported values in the literature and presented in Table 5. While some studies involving nitrogen-doped, amine-functionalized, or metal-oxide-supported biochars have achieved CO_2_ uptake values exceeding 2.0 mmol/g, such modifications often involve complex synthesis procedures, costly reagents, or multiple processing steps that reduce scalability and sustainability. In contrast, the maximum adsorption capacity of 1.58 mmol/g (35.34 cm^3^/g STP) achieved by the ZnCl_2_-activated sawdust biochar at 500 °C (S500ZC) in this study is highly promising, considering the simplicity, cost-effectiveness, and environmental friendliness of the activation route.

For instance, certain nitrogen-doped biochars derived from urea or melamine modifications have demonstrated CO_2_ uptakes around 1.8–2.3 mmol/g at 25 °C but typically require high doping levels and post-pyrolysis treatments. Similarly, KOH and NaOH-activated carbon materials have shown high microporosity and adsorption performance; however, these activating agents are corrosive and generate hazardous by-products during synthesis. The ZnCl_2_ activation employed here provides a balanced approach, offering competitive surface area development and microporosity without the need for additional surface functionalization.

Moreover, when comparing biochars derived from similar lignocellulosic biomass sources (e.g., wood chips, corn stover, bamboo), the CO_2_ adsorption capacities usually range between 0.5 and 1.5 mmol/g, depending on pyrolysis temperature and activation method. The S500ZC sample in this work not only exceeds this range but does so with a relatively moderate activation protocol and using sawdust, a widely available and low-cost precursor.

Overall, the CO_2_ adsorption study demonstrates that ZnCl_2_-activated biochars, especially those prepared at higher pyrolysis temperatures, are highly effective for CO_2_ capture. The significant difference in adsorption capacity between low- and high-temperature biochars highlights the importance of thermal treatment in tuning pore architecture. The BJH plots of the respective biochars, presented in Figure 5a–d, further substantiate the textural differences influencing the adsorption behavior. Among all, S500ZC emerges as the most promising adsorbent, offering a high surface area, narrow pore size distribution, and superior CO_2_ uptake, confirming the viability of ZnCl_2_-activated sawdust-derived biochar as a sustainable CO_2_ sorbent.

The CO_2_ adsorption capacities observed in this study are obtained under controlled low-pressure conditions (P/P_0_: 0–0.016) at 25 °C using high-purity CO_2_ gas, which is a common approach for evaluating physisorption performance and pore accessibility. Table 5 compares the adsorption capacities of the synthesized biochars with those reported in the literature. It should be noted, however, that direct numerical comparison may be limited by differences in testing conditions such as pressure, gas composition, and adsorption temperature. For example, several reported values correspond to measurements conducted at or near atmospheric pressure (1 bar), which may enhance uptake due to higher driving forces for adsorption. Furthermore, while the results presented here offer valuable insight into the intrinsic capacity of the ZnCl_2_-activated biochars, actual flue gas streams typically contain other components such as N_2_, H_2_O, and trace impurities. These species may compete with CO_2_ for adsorption sites or influence adsorption kinetics and selectivity. Future work should, therefore, include multi-component adsorption studies under more realistic conditions to comprehensively assess the material’s applicability for industrial carbon capture.

In addition to the critical role of pore structure, the presence of surface heteroatoms such as nitrogen, phosphorus, and sulfur, as confirmed by EDS and FTIR analyses, may further contribute to CO_2_ adsorption performance. Specifically, nitrogen functionalities, such as pyrrolic and amine groups are known to enhance CO_2_ adsorption via Lewis acid-base interactions and electrostatic affinity with acidic CO_2_ molecules. FTIR analysis revealed persistent peaks near 1580 cm^−1^, indicative of N–H bending. Nonetheless, such nitrogen functionalities are widely reported in the literature to improve affinity for acidic gases due to their basic character [56,57,58].

Phosphorus, detected through both EDS and minor FTIR bands, may also contribute to CO_2_ uptake by introducing surface basicity or interacting through electron-rich phosphate-like groups [59]. Sulfur species, although present in trace amounts, could modulate surface polarity or facilitate weak interactions, though their exact role remains less understood.

## 4. Conclusions

This study has systematically investigated the effects of ZnCl_2_ chemical activation and pyrolysis temperature on the physicochemical and CO_2_ adsorption properties of sawdust-derived biochars. Through comprehensive characterization, including particle size analysis, BET surface area, pore structure, and CO_2_ adsorption isotherms, the role of activation and thermal treatment in tailoring the adsorption performance is clearly elucidated. The following key conclusions summarize the major findings of this work:

1. ZnCl_2_ activation significantly enhances microporosity and surface area of sawdust-derived biochars, especially at elevated pyrolysis temperatures, making them more effective for gas adsorption applications.
2. Pyrolysis temperature plays a critical role in pore development. Samples prepared at 500 °C exhibit higher surface areas and more developed microporous structures than those produced at 300 °C.
3. The BET surface area increased from 4.12 m^2^/g (S500NZC) to 717.60 m^2^/g (S500ZC) after ZnCl_2_ activation at 500 °C, demonstrating the powerful effect of chemical activation on pore architecture.
4. Average pore diameter reduced significantly upon activation, with S500ZC achieving a narrow average pore size of 14.13 Å, ideal for CO_2_ adsorption due to its compatibility with the kinetic diameter of CO_2_ molecules (~3.3 Å).
5. CO_2_ adsorption capacity followed the trend S300NZC < S300ZC < S500NZC < S500ZC, correlating well with micropore area and BET surface area, emphasizing the dominant role of microporosity in CO_2_ physisorption.
6. A maximum CO_2_ adsorption capacity of 1.58 mmol/g (35.34 cm^3^/g STP) was achieved by the S500ZC sample, demonstrating the synergy between high pyrolysis temperature and ZnCl_2_ activation.
7. Overall, ZnCl_2_-activated sawdust biochar at 500 °C emerges as a sustainable, scalable, and efficient material for CO_2_ capture, offering an excellent balance of high surface area, narrow pore distribution, and eco-friendly synthesis.
8. ZnCl_2_-activated sawdust biochars exhibit superior CO_2_ adsorption owing to enhanced microporosity and surface functionalities. Moreover, ZnCl_2_ activation is more sustainable than KOH due to its lower corrosivity, energy demand, and easier post-treatment with potential for zinc recovery and higher biochar yield.

## Figures and Tables

**Table 1:** Particle size distribution of biochar.

Parameter		S300NZC	S300ZC	S500NZC	S500ZC
Transmittance (Red) (%)		88.4 ± 1.2	81.1 ± 1.0	60.8 ± 0.8	58.6 ± 1.1
Transmittance (Blue) (%)		88.7 ± 1.4	82.1 ± 1.1	59.5 ± 1.8	57.8 ± 1.2
Median size (μm)		11.489 ± 0.62	8.444 ± 0.48	5.704 ± 0.34	5.749 ± 0.39
Mean size (μm)		17.334 ± 0.97	9.461 ± 0.51	11.494 ± 0.65	8.052 ± 0.46
Diameter on cumulative(μm)	10 (%)	4.387 ± 0.22	4.393 ± 0.20	1.616 ± 0.12	2.208 ± 0.14
	50 (%)	11.489 ± 0.61	8.444 ± 0.45	5.704 ± 0.33	5.749 ± 0.38
	90 (%)	33.330 ± 1.78	15.299 ± 0.87	25.913 ± 1.35	14.589 ± 0.76

**Table 2:** EDS analysis of elemental distribution on biochar surfaces.

Biochar ID	Element	C	N	O	P	S	Zn	Cl
S300NZC	Mass (%)	60.1	12.7	23.8	3.2	0.2	-	-
S300ZC	Mass (%)	51.2	10.2	29.1	4.2	0.4	3.23	1.67
S500NZC	Mass (%)	65.7	15.8	15.6	2.8	0.1	-	-
S500ZC	Mass (%)	64.5	15.3	12.4	4.1	0.4	1.75	1.53

**Table 3:** N_2_-BET results.

Biochar ID	BET Surface Area (m^2^/g)	Micropore Area (m^2^/g)	BJH Pore Diameter (Å)
S300NZC	4.9574 ± 0.32	0.2089 ± 0.02	90.172 ± 5.3
S300ZC	4.1229 ± 0.26	1.3304 ± 0.05	86.059 ± 4.1
S500NZC	458.1404 ± 12.4	365.0903 ± 10.2	15.578 ± 0.45
S500ZC	717.5997 ± 14.3	616.6000 ± 13.7	14.134 ± 1.37

**Table 4:** CO_2_ adsorption volume of biochar.

Biochar ID	CO_2_ Adsorbed Volume
S300NZC	8.691
S300ZC	15.5306
S500NZC	33.696
S500ZC	35.3396

**Table 5:** Comparison of surface areas and CO_2_ adsorption capacities of biochars made from different feedstocks.

No	Feedstock	Activation	Pyrolysis at (°C)	Post Surface Treatment	Surface Area (m^2^/g)	CO_2_ Intake at 25 °C (mmol/g)	Surface Features	Ref.
1.	Korean oak	-	400	-		0.597	-	[45]
2.	Soybean stover	-	700	-	-	0.707	-	[45]
3.	Japanese oak	-	500	-	-	0.379	-	[45]
4.	Rice husk	HF	830	N_2_& ammonia at 600 °C	451.02	1.8	5.03 wt.% N	[46]
5.	Cotton stalk	KOH	600	N_2_& ammonia at 700 °C	297	1.1	Amine groups	[47]
6.	Arundo donax	Chitosan/ZnCl_2_	500	-	1863	2.1	3.91 wt.% N	[48]
7.	Walnut shell	Mg(NO_3_)_2_ 6H_2_O	900	Heating in N_2_ at 500 °C	292	1.9	Amine groups	[49]
8.	Whitewood	Mg(NO_3_)_2_ 6H_2_O	500	Steam activation	615	1.1	5.41 wt.% N	[50]
9.	Africa palm shells	KOH	600	-	365	1.9	Ultra micropores	[51]
10.	Vine shoots	None	600	In CO_2_ for 3 h at 800 °C	767	1.58	-	[52]
11.	Vine shoots	KOH: H_2_O (5:1)	600	Heating at 700 °C for 1 h	1439	1.98	Presence of N	[52]
12.	Whitewood	Steam	500	-	840	1.34	Presence of N	[53]
13.	Whitewood	CO_2_	500	-	820	1.43	Presence of N	[53]
14.	Whitewood	KOH	500	-	1400	1.77	Micro porosity	[53]
15.	Bamboo stem	None	500	-	9.72	1.01	Micro porosity	[13]
16.	Orange peel	None	500	-	51.63	0.63	Presence of amine functional groups	[13]
17.	Soybean	ZnCl_2_	600	CO_2_ Physical activation	811	0.93	Presence of N	[54]
18.	Bagasse	ZnCl_2_	500	-	923	1.74	-	[55]
19.	Rice husk	ZnCl_2_	500	-	927	1.29	-	[55]
20.	Sawdust	None	300	-	4.95	0.39	Presence of N	Present study
21.	Sawdust	None	500	-	4.12	0.69	Presence of N	Present study
22.	Sawdust	ZnCl_2_	300	-	458.14	1.50	Presence of O-functional groups	Present study
23.	Sawdust	ZnCl_2_	500	-	717.60	1.58	Presence of O-functional groups	Present study

## Data Availability

The original contributions presented in this study are included in the article. Further inquiries can be directed to the corresponding authors.

## Abbreviations

Å	Ångström (10^−10^ m)
CO_2_	Carbon Dioxide
°C	Degree Celsius
BET	Brunauer–Emmett–Teller
BJH	Barrett–Joyner–Halenda
EDS	Energy-Dispersive X-ray Spectroscopy
FTIR	Fourier-Transform Infrared Spectroscopy
SEM	Scanning Electron Microscopy
STP	Standard Temperature and Pressure
NZC	Non-ZnCl_2_-Activated Biochar
ZC	ZnCl_2_-Activated Biochar
S300NZC	Sawdust biochar, 300 °C, non-activated
S300ZC	Sawdust biochar, 300 °C, ZnCl_2_-activated
S500NZC	Sawdust biochar, 500 °C, non-activated
S500ZC	Sawdust biochar, 500 °C, ZnCl_2_-activated
P/P_0_	Relative Pressure
µm	Micrometer
